# Circulating Oxidative Stress Biomarkers in Clinical Studies on Type 2 Diabetes and Its Complications

**DOI:** 10.1155/2019/5953685

**Published:** 2019-05-12

**Authors:** Elisabetta Bigagli, Maura Lodovici

**Affiliations:** Department of Neuroscience, Psychology, Drug Research and Child Health-NEUROFARBA–Section of Pharmacology and Toxicology, University of Florence, Viale Gaetano Pieraccini 6, Florence, Italy

## Abstract

Type 2 diabetes (T2DM) and its complications constitute a major worldwide public health problem, with high rates of morbidity and mortality. Biomarkers for predicting the occurrence and development of the disease may therefore offer benefits in terms of early diagnosis and intervention. This review provides an overview of human studies on circulating biomarkers of oxidative stress and antioxidant defence systems and discusses their usefulness from a clinical perspective. Most case-control studies documented an increase in biomarkers of oxidative lipid, protein, and nucleic acid damage in patients with prediabetes and in those with a diagnosis of T2DM compared to controls, and similar findings were reported in T2DM with micro- and macrovascular complications compared to those without. The inconsistence of the results regarding antioxidant defence systems renders difficulty to draw a general conclusion. The clinical relevance of biomarkers of oxidative lipid and protein damage for T2DM progression is uncertain, but prospective studies suggest that markers of oxidative nucleic acid damage such as 8-hydroxy-2′-deoxyguanosine and 8-hydroxyguanosine are promising for predicting macrovascular complications of T2DM. Emerging evidence also points out the relationship between serum PON1 and serum HO1 in T2DM and its complications. Overall, enhanced oxidative damage represents an underlying mechanism of glucose toxicity in T2DM and its related micro- and macrovascular complications suggesting that it may be considered as a potential additional target for pharmacotherapy. Therefore, further studies are needed to understand whether targeting oxidative stress may yield clinical benefits. In this view, the measurement of oxidative stress biomarkers in clinical trials deserves to be considered as an additional tool to currently used parameters to facilitate a more individualized treatment of T2DM in terms of drug choice and patient selection.

## 1. Introduction

Type 2 diabetes (T2DM) and its complications constitute a major worldwide public health problem, with high rates of morbidity and mortality [1]. T2DM is strongly associated with both microvascular (retinopathy, nephropathy, and neuropathy) and macrovascular complications, including ischemic heart disease, peripheral vascular disease, and stroke [2]. Since T2DM often remains undiagnosed due to the mild or asymptomatic nature of this condition, vascular complications may be already present in the early phases of the disease and even in the prediabetic stage [3]. Biomarkers predictive of the occurrence and development of T2DM and its complications may therefore offer benefits in terms of early diagnosis and intervention, thus slowing down disease progression. Oxidative stress, defined as an imbalance between the production of reactive oxygen species (ROS) and antioxidant defence systems, has been often associated with the development of diabetes and its complications [4–6].

Several biomarkers of oxidative stress are available, including ROS themselves. However, since ROS are very reactive and have a short half-life, it is more suitable to estimate oxidative stress by measuring their oxidation target products, including lipid peroxidation, oxidized proteins, and oxidative nucleic acid damage [7].

In this review, we will provide an overview of human studies on circulating biomarkers of ROS-induced modifications of lipids, nucleic acids, and proteins as well as markers of antioxidant defence systems evaluated in the plasma, serum, or urines of T2DM patients and discuss their utility in predicting the onset and progression of the disease.

## 2. Overview on the Mechanisms of Oxidative Stress Generation and Antioxidant Defence Systems

**Figure 1:**
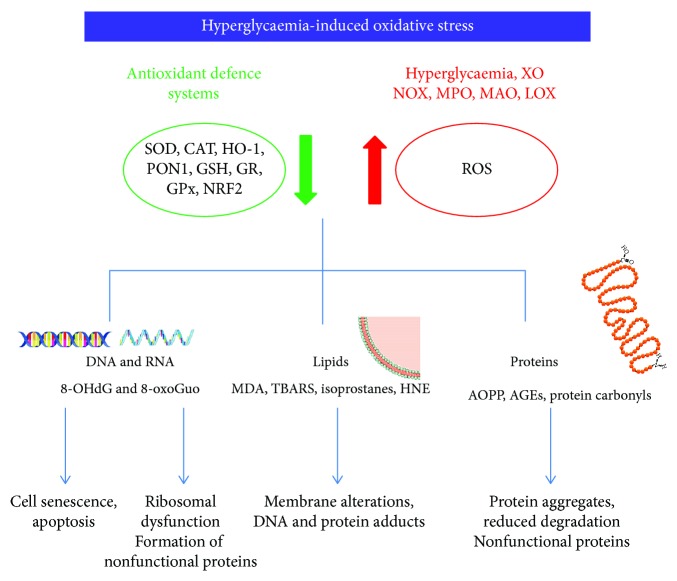
Mechanisms of hyperglycaemia-induced oxidative damage to nucleic acids, lipids and proteins. All abbreviations are spelled out in the text.

ROS including superoxide (O_2_^·-^), hydroxyl radical (OH^·^), hydrogen peroxide (H_2_O_2_), and singlet oxygen (^1^O_2_^−^) are generated during normal aerobic metabolism, and low levels are necessary for several basic biological processes including cellular proliferation and differentiation [8, 9]. Many cell types can produce ROS including macrophages, neutrophils, and endothelial and epithelial cells; however, excessive production can induce oxidative stress with detrimental effects on cellular components such as nucleic acids, proteins, and lipids [10–13] (Figure 1).

### 2.1. Sources of Oxidative Stress

Mitochondria have a major contribution to ROS production, particularly O_2_^·-^, a side product of electron transport during oxidative phosphorylation. H_2_O_2_ is produced *in vivo* by many reactions, easily crosses cellular membranes and, receiving one or more electrons from iron or copper, generates OH^·^, the most abundant and damaging radical in the body, although very short-lived [14].

The two major ROS-generating enzymatic systems are nicotinamide adenine dinucleotide phosphate (NADPH) oxidase (NOX family) and xanthine oxidase (XO). Although the expression of NOX was initially thought to be confined to neutrophils and macrophages, it is also present in endothelial cells, cardiomyocytes, hematopoietic stem cells, and platelets [15]. During purine degradation, XO catalyses electron transfer to oxygen molecules which generates H_2_O_2_ and O_2_^·-^ species [16]. Although with minor activity, other sources of ROS include myeloperoxidase (MPO), which contributes to the progression of atherosclerotic plaque by oxidizing LDL [15], lipoxygenase (LOX), cyclooxygenase (COX), and monoamine oxidases (MAO-A and MAO-B) [17]. Endothelial nitric oxide synthase (eNOS) normally produces the potent vasodilator NO^·^ by catalysing the conversion of L-arginine to L-citrulline, and in this process, the presence of cofactor tetrahydrobiopterin (BH_4_) is essential [18]. However, in the absence of either L-arginine or BH_4_, eNOS can produce O_2_^·-^, in a phenomenon referred as “eNOS uncoupling” associated with increased ROS production in aging and cardiovascular diseases [18] (Figure 1).

### 2.2. Enzymatic and Nonenzymatic Antioxidant Defence Systems

The enzymatic and nonenzymatic antioxidant defence systems regulate ROS formation and protect biological systems from ROS-induced oxidative damage. Among the antioxidant enzymes, superoxide dismutase (SOD), catalase (CAT), glutathione peroxidase (GPx), and paraoxonase (PON) are the most studied. The nonenzymatic sources of antioxidants include ascorbic acid, tocopherol, uric acid, and glutathione (GSH); the latter acts as a ROS scavenger, and it is the substrate of the GPx enzyme; during this reaction, GSH is oxidized to GSSG, and through glutathione reductase (GR) activity, it is reconverted to GSH with NADPH as cofactor. SOD catalyses the conversion of O_2_^·-^ to H_2_O_2_ that can be converted to H_2_O through the CAT enzyme [19] or through GSH by GPx [20]. PONs are a family of three enzymes able to hydrolyse aryl esters, lactones, and organophosphates, synthesized mainly in the liver and secreted into the plasma. PON1 is involved in lipid metabolism since it is a key functional constituent of HDL and protects low-density lipoprotein (LDL) from oxidation [21]. Among antioxidant protective systems, Nrf2 (nuclear factor-E2-related factor 2) is a transcription factor capable to induce a set of antioxidant and detoxification enzymes [22]. Under stressful conditions or in the presence of Nrf2-activating compounds, Nrf2 translocates to the nucleus to induce the expression of its target genes such as heme oxygenases (HOs), a family of enzymes that catalyses the degradation of heme producing biliverdin, ferrous iron, and carbon monoxide [23]. HO1 is localized in the brain, kidney, heart, liver, and vascular smooth muscle cells, and its induction is important in initiating protective mechanisms in response to stressful stimuli [24] (Figure 1).

## 3. Association between Oxidative Damage and Antioxidant Defence Systems with the Development of T2DM and Its Complications

**Figure 2:**
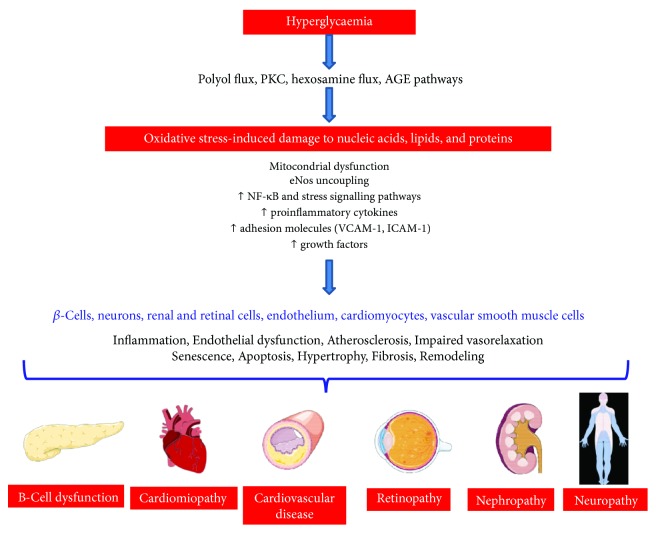
Schematic diagram summarizing the damaging effects of oxidative stress-induced damage to nucleic acids, lipids, and proteins leading to the development and progression of diabetic complications including cardiomyopathy and cardiovascular diseases, retinopathy, nephropathy, and neuropathy. All abbreviations are spelled out in the text.

Chronic hyperglycaemia leads to the generation of oxidative stress in pancreatic *β*-cells which are particularly vulnerable to the damaging effects of excessive ROS production because of their lower abundance of antioxidant defence enzymes, compared to other tissues [25]. Due to their ability to directly damage and oxidize DNA, protein and lipid ROS lead to *β*-cell dysfunction and death. In addition to macromolecular damage, ROS can activate a number of cellular stress-sensitive pathways that have been linked to insulin resistance and decreased insulin secretion [6]. The development of not only *β*-cell dysfunction and insulin resistance but also the late complications of diabetes has been linked to hyperglycaemia-induced oxidative stress through the four main molecular mechanisms: the polyol pathway, advanced glycation end product (AGE) formation, the protein kinase C- (PKC-) diacylglycerol (DAG), and the hexosamine pathways [26, 27]. Moreover, oxidative stress originates from the oxidative biochemistry of glucose itself, which undergoes autoxidation generating ROS which in turn can directly oxidize and damage DNA, RNA, proteins, and lipids activating a number of cellular stress-sensitive pathways that cause cellular damage in the endothelial cells of large and small vessels as well as in the myocardium engaging vicious cycles that further exacerbate organ dysfunction [5]. In fact, oxidative stress is associated with increased expression of proinflammatory cytokines, growth factors, procoagulant factors, adhesion molecules, and decreased nitric oxide release, all pathophysiological events leading to endothelial dysfunction and micro- and macrovascular diseases [28, 29] (Figure 2).

### 3.1. AGEs in the Development of T2DM and Its Complications

In T2DM, the presence of persistently elevated glucose levels increases the frequency of AGEs, a heterogeneous group of compounds derived from the nonenzymatic glycation of proteins, lipids, and nucleic acids through the Maillard reaction [30]. Glycation, in addition to glycoxidation, can cause structural and functional impairments of plasma proteins in particular albumin [31], and it is involved in the pathophysiological mechanism of vascular diseases in T2DM [32]. By binding to their cell surface receptor (RAGE) on macrophages and endothelial cells, AGEs trigger a cascade of ROS generation and activation of proinflammatory pathways and profibrotic factors such as nuclear factor-*κ*B (NF-*κ*B), vascular cell adhesion molecule-1 (VCAM-1), intercellular adhesion molecule-1 (ICAM-1), plasminogen activator inhibitor-1 (PAI-1), and monocyte chemoattractant protein-1 (MCP-1) involved in the progression of atherosclerosis and vascular pathology [33]. Immunohistochemical studies have shown that AGEs accumulate in the mesangium and glomerular capillary wall [34] and in the peripheral nerves of diabetic patients [35] suggesting their role in the pathogenesis of microvascular complications.

### 3.2. Lipid Peroxidation in the Development of T2DM and Its Complications

Bioactive products of lipid peroxidation induce disturbances in membrane organization, functional loss, and modifications of enzymes, carriers, and cytoskeletal and mitochondrial proteins as well as DNA bases leading to cell death or inducing alterations in the biochemical properties of these biomolecules [36].

### 3.3. Protein Oxidation in the Development of T2DM and Its Complications

The oxidative modification of proteins has been also linked to the pathological conditions of the vascular system: the most well-studied proteins are carbonylated proteins which accumulate as nonfunctional protein aggregates that can become cytotoxic [7].

Advanced oxidation protein products (AOPPs) are dityrosine-containing and crosslinking proteins found in atherosclerotic lesions and promote vascular inflammation, monocyte activation, and endothelial dysfunction through the overexpression of adhesion molecules such as ICAM-1 and VCAM-1 [37, 38].

### 3.4. Nucleic Acid Oxidative Damage in the Development of T2DM and Its Complications

Oxidative stress also leads to DNA damage and accumulation of 8-hydroxy-2′-deoxyguanosine (8-OHdG) in atherosclerotic plaques [39] as well as in the kidney of diabetic rats [40]. Another possible mechanism by which DNA oxidation could be involved in the development of vasculopathy and atherosclerosis is the induction of cell senescence via telomeric or nontelomeric DNA damage [41]. RNA also undergoes significant oxidation forming 8-hydroxyguanosine (8-oxoGuo), which leads to ribosomal dysfunction, formation of nonfunctional or truncated proteins, and reduced levels of functional proteins [42]. RNA and DNA oxidation appears in the early stage of diabetic nephropathy [43], and the levels of oxidized purines and pyrimidines are elevated in the iris tissues of diabetic patients with glaucoma [44].

### 3.5. Antioxidant Defence System in the Development of T2DM and Its Complications

A defective antioxidant defence system has been also associated with diabetes and its complications: because of their low levels of antioxidants, *β*-cells are particularly susceptible to ROS, and hyperglycaemia itself depletes GSH by causing nonenzymatic glycation of GR and GPx, with inhibition of their reductive enzymatic activity [45]. Decreased SOD activity has been implicated in the pathogenesis of retinopathy in diabetes, and its overexpression prevented hyperglycaemia-associated production of ROS, activation of PKC, and AGE formation [46, 47]. There is also evidence that HO1 can protect against vascular damage and atherogenesis [48]: HO1 is upregulated in macrophages during the development of inflammation in atherosclerosis, with a consequently decreased expression of VCAM-1 and proinflammatory cytokines [49]; HO1 overexpression inhibits atherosclerosis by reducing ox-LDL both in the plasma and in the artery wall and reduces glomerular injury and apoptosis in diabetic rats [50–52]. PON1 also has an atheroprotective function, and its reduced activity in T2DM seems to be due to its nonenzymatic glycation [53].

## 4. Biomarkers of Lipid Peroxidation

Lipid peroxidation is the free radical oxidation of polyunsaturated fatty acids (PUFAs) such as linoleic acid or arachidonic acid, and it is capable of extensive tissue damage [54]. ROS-induced peroxidation of membrane lipids, in fact, alters the structure and the fluidity of biological membranes, which ultimately affect their function. Among the most frequently studied markers of lipid peroxidation are isoprostanes such as 8-iso-prostaglandin F2*α* (8-iso-PGF2*α*), malondialdehyde (MDA), thiobarbituric acid reactive substances (TBARS), and hydroxynonenal (HNE) [55]. MDA is a highly reactive nucleophilic agent generated by both lipid peroxidation and as a by-product of prostaglandin and thromboxane synthesis that can attack macromolecules, including amino acid or sulfhydryl moiety of proteins leading to alterations in their functions [56]. HNE is a major toxic aldehyde generated by ROS attack to *ω*-6 polyunsaturated fatty acids and reacts with proteins forming advanced lipoxidation end products. Both HNE and MDA adducts were detected in atherosclerotic lesions [7].

Another lipid peroxidation product with prostaglandin-like structure, produced primarily from esterified arachidonic acid by nonenzymatic reactions catalysed by free radicals, is 8-iso-PGF2*α* [55] that can contribute to platelet activation [36].

### 4.1. Lipid Peroxidation in the Prediabetic Stage (Retrospective Studies)

In prediabetic patients, urinary 8-iso-PGF2*α* were higher than in healthy volunteers but negatively correlated with HbA1c [57]. Similarly, the levels of 8-iso-PGF2*α* and MDA were higher in the plasma of prediabetics compared to healthy subjects but lower than in the T2DM patients [58]. However, other studies reported no differences in urinary isoprostanes and TBARS between prediabetes and controls [59, 60] (Table 1).

### 4.2. Lipid Peroxidation in T2DM with and without Complications (Retrospective Studies)

One of the most consistent findings on lipid peroxidation markers in T2DM patients with and without complications was a significant increase in TBARS or MDA compared to healthy controls [61] (Table 1) and in T2DM patients with micro- and macrovascular complications as compared to those without [62–64] (Table 2). MDA was also significantly increased in T2DM both with and without complications and performed better than ischemia-modified albumin (IMA), but it was of minor value compared to glycated haemoglobin (HbA1c) measurement in the evaluation of diabetes progression [65] (Table 2). T2DM patients with poor glycaemic control had significantly higher levels of MDA, when compared with the controlled T2DM patients and the control group [66, 67] (Tables 1 and 2). We also previously reported that circulating MDA was increased in poorly controlled T2DM with and without complications [63], and this effect was more pronounced in females [68]; however, other authors found no differences in MDA and isoprostanes between female T2DM patients with high or low HbA1c [69] (Table 2).

Significantly higher levels of MDA in T2DM with ischemic heart disease were found in the study of Djindjic et al. [70] and in T2DM with chronic kidney disease as compared to patients without complications and healthy controls [71] whereas other studies reported no differences in patients with or without nephropathy [72, 73] (Table 2).

## 5. Advanced Glycation End Products (AGEs)

AGEs are a complex group of compounds formed via a nonenzymatic reaction between reducing sugars and amine residues on proteins, lipids, or nucleic acids. The major AGEs in vivo appear to be formed from highly reactive intermediate carbonyl groups, including 3-deoxyglucosone, glyoxal, and methylglyoxal [74]. Some of the best chemically characterized AGEs in humans include pentosidine and N(carboxymethyl)lysine [75].

### 5.1. AGEs in T2DM with and without Complications (Retrospective Studies)

Plasma AGE levels were significantly higher in T2DM compared to controls and in diabetics with vascular complications compared to those without [64, 76, 77] (Table 2). On the contrary, Chou and Tseng [73] found no differences in AGE levels in T2DM with mild or severe nephropathy and those without (Table 2).

### 5.2. AGEs in Prospective Clinical Studies

Baseline plasma levels of AGEs were associated with several subclinical atherosclerosis parameters over 10 years of follow-up in patients with long-standing T2DM [78] and predicted the incidence of cardiovascular disease [79]. However, in a recent large prospective clinical trial, AGEs were not associated with the risk of major adverse cardiovascular disease in multivariate analysis [80] and were not associated with the risk of major lower extremity artery disease in T2DM [81] (Table 3).

## 6. Biomarkers of Protein Oxidation

Carbonyl derivatives (aldehydes and ketones) are formed by direct oxidation of amino acid residues by ROS or nonoxidative reactions with carbonyl-containing oxidized lipids [82]. Carbonyl levels are the most widely used marker of oxidative protein damage, because of the relative early formation and stability [83]. Advanced oxidation protein products (AOPPs) are formed in the reaction of chlorinated oxidants, such as chloramines and hypochlorous acid with plasma proteins, mostly albumin [82].

### 6.1. Protein Carbonyl and AOPPs in T2DM with and without Complications (Retrospective Studies)

Protein carbonyl and AOPPs were significantly higher in T2DM patients in comparison to healthy volunteers [62, 65, 76, 84] and were also increased in those with micro- or macrovascular complications compared to T2DM without complications [62, 64, 65, 76, 84] (Tables 1 and 2). A progressive increase in AOPP plasma levels in the course of albuminuria was also noted, and AOPP was better than IMA in distinguishing patients with micro- and macroalbuminuria [85] (Table 2). Plasma AOPP concentrations were an independent risk factor for endothelial dysfunction in individuals at an early stage of diabetes [86] (Table 2). Increased protein carbonyls in red blood cells were also observed in diabetic retinopathy [87] but not in T2DM with stable ischemic heart disease [70] (Table 2). Carbonyl residues in poorly controlled T2DM with and without complications were higher compared to well controlled without complications [63] and were positively associated with the cardiovascular risk score [88] (Table 2).

### 6.2. Protein Carbonyl and AOPPs in Prospective Clinical Studies

A recent large prospective clinical trial indicated that both carbonyls and AOPP were not associated with the risk of major adverse cardiovascular events [80]; similarly, no association between the baseline levels of protein carbonyls and lower-extremity artery disease was observed by Nativel et al. [81] (Table 3).

## 7. Biomarkers of Oxidative DNA and RNA Damage

### 7.1. 8-OHdG and 8-oxoGuo in Prediabetes and in T2DM (Retrospective Studies)

A well-known biomarker of oxidative DNA damage is 8-OHdG [89], and consistent evidence from observational studies showed increased urinary 8-OHdG levels in both prediabetes and T2DM compared to controls [57, 59, 90] (Tables 1 and 2). Urinary levels of 8-OHdG and of the RNA oxidation marker, 8-oxo-7,8-dihydroguanosine (8-oxoGuo), were elevated in T2DM patients with and without complications compared with age-matched healthy controls [91]. Jelinek et al. [92] also identified 8-OHdG as a HbA1c comarker for T2DM diagnosis.

### 7.2. 8-OHdG and 8-oxoGuo in T2DM with and without Complications (Retrospective Studies)

Patients with complications, especially macrovascular complications, exhibited higher levels of 8-OHdG than those without complications [91] (Table 2). Chou and Tseng [73] reported increased plasma-8-OHdG levels in diabetic patients with micro- and macroalbuminuria compared to normoalbuminuric patients. Similarly, urinary 8-OHdG levels were significantly higher in T2DM with microvascular complications when compared to those without and better discriminate microvascular complications compared with urinary albumin [93]. On the contrary, Serdar et al. [90] found no differences in patients with and without nephropathy (Table 2).

### 7.3. 8-OHdG and 8-oxoGuo in Prospective Clinical Studies

Urinary excretion of 8-oxoGuo measured shortly after the diagnosis of T2DM predicted long-term mortality independent of conventional risk factors [94] (Table 3). In the ADVANCE prospective trial, increased levels of 8-OHdG were independently associated with all-cause and cardiovascular mortality in 3766 T2DM [95]. In a recent prospective cohort study involving 1863 patients with T2DM, 8-oxoGuo was associated with all-cause mortality and cardiovascular death after multiple regression analysis [96]. Moreover, recent findings suggest that urinary 8-oxoGuo provides additional information about risk to that obtained from urinary albumin and that the combined use of 8-oxoGuo and urinary albumin could be useful for a better identification of patients at risk of CVD and death over a period of 19 years of follow-up [97] (Table 3).

## 8. Biomarkers of Antioxidant Defence Systems

### 8.1. Nonenzymatic Antioxidant Defence Systems (Retrospective Studies)

Reduced levels of GSH and Nrf2 [59, 67] and no change in total antioxidant status [58] were reported in the prediabetic stage compared to controls. Total antioxidant status was reduced in T2DM patients compared to controls and prediabetic patients in [58, 67, 84], increased in T2DM patients compared to controls in [98], and unchanged in [99] (Table 1). We previously reported that FRAP levels were significantly lower only in diabetics with poor glycaemic control, while patients with good glycaemic control had FRAP values similar to controls [100]; on the contrary, total antioxidant status was reduced in T2DM compared to controls, independently of glycaemic control in [67] (Table 1).

Reduced GSH [67] was observed in T2DM with good and poor glycaemic control compared to controls [61, 67, 99], whereas Nrf2 was reduced only in T2DM with poor glycaemic control [67] (Table 1). Grindel et al. [69] found no differences in GSH and FRAP between T2DM female patients with and without good glycaemic control. Comparing T2DM patient with complication to those without, decreased GSH and FRAP [62, 71] and vitamin C [73] increase in FRAP levels [68], and no differences in GSH and total antioxidant status were reported [73] (Table 2). Recently, in a large prospective clinical trial, the total antioxidant capacity of plasma was not associated with the risk of major cardiovascular events [80] (Table 3).

### 8.2. Enzymatic Antioxidant Defence Systems (Retrospective and Prospective Studies)

Regarding antioxidant enzymatic defence systems, SOD activity was reduced [58] or unchanged [67, 70] in prediabetes compared to controls and either increased [60, 67] or decreased [58, 61] in T2DM compared to controls. No differences in CAT and GPx activity were observed between prediabetes and control in [60] (Table 1). GPx was increased in uncontrolled T2DM compared to prediabetes [67] and decreased compared to controls [98, 99] (Table 1).

Inconsistent results on SOD, CAT, and GPx were also found when T2DM patients with complications were compared to those without complications [62, 63, 68, 69, 72, 73] (Table 2). Regarding the protective antioxidant defence of GPx, González de Vega et al. [101] found the presence of glycated GPx in sera from diabetic patients, with lower GPx activity than that measured in healthy individuals, and this reduction was greater in patients with higher HbA1c.

PON1 levels decreased in T2DM patients compared to controls and correlated to the duration of diabetes [102–106] (Table 1). PON1 was reduced in T2DM with retinopathy and macrovascular disease compared to those without [105, 106] and predicted cardiovascular events in T2DM [107] (Table 3). Lower PON1 activity was associated with an increased risk of developing T2DM in a longitudinal study with a 20-year follow-up [108]; on the contrary, in the PREVEND prospective study involving 5947 participants, no association was found [109] (Table 3). Plasma HO1 concentrations were significantly increased in new-T2DM patients compared to controls and correlated with plasma glucose levels [110] (Table 1); increased urine HO1 levels were detected in T2DM before the onset of significant albuminuria and were associated with renal derangement in patients with established diabetic nephropathy [111] (Table 2).

## 9. Discussion

Several oxidative stress biomarkers in the form of lipid, protein, and nucleic acid oxidation products have been studied in T2DM patients, while fewer data in the prediabetic stage are currently available. In the majority of the studies, urinary and plasma levels of 8-iso-PGF2*α* and urinary levels of 8-OHdG were elevated in the prediabetic stage representing potential early disease markers in patients at risk for T2DM which may also enable therapeutic interventions from the early stages of diabetes where cardiovascular risk is already increased. Similarly, when oxidative stress biomarkers were evaluated in patients with clinical diagnosis of T2DM, the most consistent finding across many of these studies was a significant increase in the plasma levels of TBARS, MDA, AGEs, protein carbonyls, and AOPP and in the urinary levels of 8-OHdG in T2DM patients compared to healthy controls; similar results were observed in T2DM patients with micro-and macrovascular complications in comparison to those without complications.

These studies present some limitations that should be addressed in future research: in particular, neither potential gender-related differences nor racial disparities in oxidative stress biomarkers have been actively investigated so far; nevertheless, both racial and gender differences in the markers of oxidative stress have been reported [68, 112, 113]. A limitation of many studies is that medications such as statins, *β*-blockers, angiotensin-converting enzyme inhibitors, and angiotensin receptor blockers which exhibit antioxidant effects were not always analysed as potential confounding factors [114]; a limitation of many studies is that these medications were not always included in the analyses as potential confounding factors.

From an overall point of view, despite the heterogeneity of T2DM patients in terms of stage and duration and the presence of different complications, what emerges from retrospective human studies is that there is an association between increased circulating oxidative damage biomarkers, T2DM, and its complications. Nevertheless, none of these biomarkers has never reached clinical practice mainly because of the lack of standardized methods and reference range intervals and the lack of validation in prospective trials. In fact, in spite of the promising results from observational clinical studies, a recent large prospective study concluded that none of the different biomarkers of oxidative stress used (AGE, AOPP, protein carbonyls, and antioxidant capacity of plasma) was associated with adverse cardiovascular events [80]. However, encouraging results were obtained with the nucleic acid oxidation biomarkers, 8-oxoGuo and 8-OHdG measured in the urines, which may be useful to predict micro- and macrovascular complications of T2DM. Data referring to antioxidant defence systems are more inconclusive since they have been found either decreased or increased. Emerging evidence also points out the relationship between serum PON1 and HO1 in T2DM and its complications, and a recent meta-analysis demonstrated that PON1 was significantly associated with the susceptibility of T2DM and to the development of macro- and microangiopathy [115].

From a mechanistic point of view, even if oxidative stress is involved in nearly all stages of micro- and macrovascular disease development [116–118], the relative importance of each biomarker of oxidative nucleic, protein, or lipid damage and whether one may be related to a greater extent with the key mechanisms of T2DM and its complications need further investigations (Figure 2).

The evaluation of oxidative damage biomarkers may be also relevant for better phenotyping patients; Seddon et al. [116] highlighted that oxidative stress is a redox disease and a potential covariate in predicting patient recovery on an antidiabetic treatment; this highlights the need to identify those patients who are likely to need specific pharmacological strategies that can target oxidative stress, beyond lowering glucose.

Moreover, several drugs used for the treatment of T2DM have been shown to exert pleiotropic effects [117] but, to date, the relationship between glycaemic control, oxidative stress, and endogenous antioxidant defence systems has received limited attention in clinical studies.

In particular, it is the case of the newer glucose-lowering drugs such as GLP-1 analogues, dipeptidyl peptidase 4 (DPP4) inhibitors, and sodium-glucose cotransporter 2 inhibitors (SGLT2i). Interestingly, a recent large study demonstrated that GLP-1 analogues, beyond improving glycaemic control and promoting weight loss, improve cardiovascular outcomes in patients at high cardiovascular risk [118], and this effect was associated with reduced oxidative stress [119].

## 10. Conclusions

There is a need to improve our knowledge about T2DM and its complications in order to be able to develop new targets of intervention. Oxidative stress represents one of the mechanisms involved in the pathogenesis and progression of T2DM and its complications, suggesting that it may be considered as an additional target for pharmacotherapy. In this view, the measurement of the circulating biomarkers of oxidative stress offers several advantages including easy collection, low cost, and the possibility of use in large clinical studies. At present, among several biomarkers, urinary 8-OHdG and 8-oxoGuo, serum PON1 and HO1 seem to be the most promising for predicting cardiovascular disease in T2DM.

However, the measurement of oxidative stress biomarkers may have several other clinical applications such as patient selection and stratification as well as monitoring the clinical efficacy of medications supposed to affect oxidative stress. Direction for future research should also include further mechanistic studies linking each biomarker to disease onset and progression including their temporal variations from the early to the late stages of the disease.

By better targeting the mechanisms involved in disease pathogenesis and progression, with old or new therapeutic strategies, we can expect to improve the outcomes of T2DM patients. Therefore, the measurement of oxidative stress biomarkers in clinical trials deserves to be considered as an additional tool to currently used parameters to facilitate a more individualized treatment of T2DM in terms of drug choice and patient selection.

## Figures and Tables

**Table 1:** Selected clinical retrospective studies on circulating oxidative stress markers in T2DM and prediabetes. All the studies included in this table were categorized under the class of evidence C, retrospective studies.

Disease and population	Sample	Markers	Observation	Information on medication or supplements	Reference
Prediabetics (*N* = 47) T2DM (*N* = 43) Controls (*N* = 37)	Urine	8-OHdG 8-iso-PGF2*α*	↑ 8-iso-PGF2*α* and 8-OHdG in prediabetes compared to controls ↑ 8-OHdG in T2DM patients compared to controls	No medication No special diet No supplements	[57]
Prediabetics (*N* = 176) Controls (*N* = 252)	Urine Erythrocytes	8-iso-PGF2*α* 8-OHdG GSH/GSSG	No differences in 8-iso-PGF2*α* ↑ 8-OHdG in prediabetes group compared to control ↓ GSH/GSSG in prediabetes compared to control	Antihypertensive drugs: Prediabetes (35.8%) Controls (5.2%) Statins and anticoagulants: Controls (<4%) Prediabetes (<16%)	[59]
Prediabetics (*N* = 49) T2DM (*N* = 30) Controls (*N* = 30)	Plasma	8-iso-PGF2*α* MDA TAOC SOD	↑ 8-iso-PGF2*α* and MDA in prediabetes and T2DM compared to controls ↑ 8-iso-PGF2*α* in T2DM compared to prediabetes ↓ TAOC in T2DM compared to controls and prediabetes ↓ SOD in prediabetes compared to controls No differences in TAOC between prediabetes and controls ↓ SOD in T2DM compared to controls and prediabetes	No use of medication that affects glucose metabolism within 6 months	[58]
Prediabetics (*N* = 111) T2DM (*N* = 186) Controls (*N* = 259)	Plasma and erythrocyte	Nrf2 MDA TAS GPx SOD GSH	↓ TAS and GSH in controlled and uncontrolled T2DM compared to controls and prediabetes ↓ Nrf2 in prediabetes and in uncontrolled T2DM compared to controls ↑ MDA in uncontrolled T2DM compared to controls and prediabetes and in controlled T2DM compared to controls ↑ GPx in uncontrolled T2DM compared to prediabetes ↑ SOD in controlled and uncontrolled T2DM compared to prediabetes and controls	Not provided	[67]
Prediabetics (*N* = 9) T2DM (*N* = 55) Controls (*N* = 29) Ethnicity: whites, blacks, Brazilians	Plasma and erythrocyte	SOD CAT GPx TBARS	No differences in TBARS, SOD, GPx, and CAT between prediabetes and controls ↑ SOD and TBARS in T2DM compared to controls and prediabetes	Antihypertensive drugs: 76.4% of T2DM, 66.7% of the pre-DM, and 37.9% of the controls Among T2DM: 92.7% sulphonylurea and/or biguanide	[60]
T2DM (*N* = 215) Controls (*N* = 37)	Serum	SOD TBARS GSH	↓ SOD and GSH in T2DM compared with controls ↑ TBARS in T2DM compared with controls	Not provided	[61]
T2DM (*N* = 39) Controls (*N* = 18)	Plasma	FRAP	↓ FRAP lower in T2DM with poor glycaemic control than controls	T2DM treated with metformin and glibenclamide in combination with other	[99]
T2DM (*N* = 31) Controls (*N* = 31)	Plasma	Protein carbonyl AOPP Radical scavenging capacity of plasma	↑ protein carbonyl content and AOPPs in T2DM in comparison to healthy volunteers ↓ Radical scavenging capacity of plasma in T2DM than controls	Not provided	[84]
T2DM (*N* = 80) Controls (*N* = 79)	Plasma	FRAP GSH GR GPx	↑ FRAP and GR in T2DM compared to controls ↓ GPx activity in T2DM compared to controls	Not provided	[98]
T2DM (*N* = 115) Controls (*N* = 32)	Plasma	GSH GPx TAS	↓ GSH, GPx in T2DM compared to controls No differences in TAS	No use of vitamins, minerals, or other supplements	[100]
T2DM (*N* = 420) Controls (*N* = 429)	Plasma	HO1	↑ HO1 in T2DM compared to controls	Not provided	[110]
T2DM (*N* = 30) Controls (*N* = 20) Ethnicity: Indian	Serum	PON1	↑ PON1 in controls compared to T2DM	Not provided	[102]
T2DM (*N* = 90) Controls (*N* = 90)	Serum	PON1	↑ PON1 in controls compared to T2DM	Not provided	[103]
T2DM (*N* = 145) Controls (*N* = 574)	Serum	PON1	↑ PON1 in controls compared to T2DM	10 controls and 15% of T2DM were on active lipid-lowering treatment	[104]

8-iso-PGF2*α*: 8-iso- prostaglandin F2*α*; 8-OHdG: 8-hydroxy-2′-deoxyguanosine; AGEs: advanced glycation end products; AOPP: advanced oxidation protein products; CAT: catalase; FRAP: ferric reducing ability of plasma; GSH: reduced glutathione; GSSG: oxidized glutathione; GPx: glutathione peroxidase; GR: glutathione reductase; HNE: 4- hydroxy-2-nonenal; HO1: heme oxygenase; MDA: malondialdehyde; Nrf2: nuclear factor erythroid 2; PON1: paraoxonase 1; SOD: superoxide dismutase; TBARS: thiobarbituric acid reactive substances; TAS: total antioxidant status.

**Table 2:** Selected clinical retrospective studies on circulating oxidative stress markers in T2DM with and without complications. All the studies included in this table were categorized under the class of evidence C, retrospective studies.

Disease and population	Sample	Markers	Observation	Information on medication or supplements	Reference
T2DM (*N* = 85) T2DM with micro- and macrovascular complications (*N* = 75) Controls (*N* = 60)	Plasma and erythrocytes	Protein carbonyl TBARS FRAP GSH CAT	↑ protein carbonyls in T2DM and T2DM with complications compared to controls and in T2DM with complications compared to T2DM without ↑ TBARS higher in T2DM and T2DM with complications compared to controls and in T2DM with complications compared to T2DM without ↓ FRAP, GSH, and CAT in T2DM and T2DM with complications compared to controls ↓ GSH in T2DM and T2DM with complications compared to controls and in T2DM with complications compared to T2DM without	No antioxidants	[62]
T2DM with complications (*N* = 50) T2DM without complications (*N* = 50) Controls (*N* = 50)	Plasma	MDA AOPP	↑ MDA and AOPP in T2DM compared to controls ↑ MDA and AOPP in T2DM with complications compared to T2DM without complications	4% of T2DM without complications and 42% of T2DM with complications were on insulin treatment 96% of T2DM without complications and 58% of T2DM with complications were on oral hypoglycaemic drug treatment	[65]
T2DM with poor glycaemic control without complications (*N* = 52) T2DM with poor glycaemic control with complications (*N* = 37)	Plasma	MDA Protein carbonyl FRAP SOD	↑ SOD and FRAP in patients with complications ↑ MDA in females with complications compared to those without No differences in carbonyl residues between males and females	Multiregression analysis: Statin treatment was associated with SOD in females Metformin treatment was inversely associated with MDA	[68]
T2DM with good glycaemic control without complications (*N* = 15) T2DM with poor glycaemic control without complications (*N* = 28) T2DM with poor glycaemic control with micro- and macrovascular complications (*N* = 29)	Plasma	MDA Protein carbonyl FRAP SOD	↑ MDA and carbonyl residues in T2DM with poor glycaemic control with and without complications compared to poorly controlled ↓ FRAP in poorly controlled without complications compared to well controlled without complications ↑ SOD in T2DM with poor glycaemic control with complications compared to well controlled	Medications (nonexclusive): sulphonylureas (*n* = 12), biguanides (*n* = 46), insulin (*n* = 30), thiazolidinediones (*n* = 2), meglitinides (*n* = 4), statins (*n* = 31), and antihypertensive drugs (*n* = 58) Multiregression analysis indicated no confounding effect of statin or metformin	[63]
T2DM with nephropathy (*N* = 50) T2DM without nephropathy (*N* = 50) Controls (*N* = 50)	Plasma	MDA FRAP GSH	↑ MDA in T2DM with nephropathy compared to those without and controls ↓ FRAP and GSH in T2DM with and without nephropathy compared to controls	Patients on inhibitors of the renin-angiotensin-aldosterone system, aspirin, and vitamin D analogues were advised to stop these drugs for one week before inclusion in the study	[71]
T2DM with nephropathy (*N* = 32) T2DM without nephropathy (*N* = 20) Controls (*N* = 20)	Urine	8-OHdG	↑ 8-OHdG in T2DM with and without nephropathy compared to controls No differences between T2DM with and without nephropathy	Not provided	[90]
T2DM patients with good glycaemic control (*N* = 27) T2DM patients with poor glycaemic control (*N* = 36) Controls (*N* = 16)	Serum	MDA	↑ MDA in T2DM with poor glycaemic control vs. good glycaemic control and healthy volunteers	No antioxidants supplementation in the previous two months	[66]
T2DM female patients with good glycaemic control (*N* = 74) T2DM female patients with poor glycaemic control (*N* = 72)	Serum, plasma, PBMC	MDA F2-isoprostanes FRAP GSH/GSSG SOD CAT GPx	No differences	Similar distribution of metformin, insulin, and other antidiabetic medications in the two groups	[69]
T2DM without nephropathy (*N* = 22) T2DM with mild nephropathy (*N* = 22) T2DM with severe nephropathy (*N* = 15)	Plasma and RBC	AGEs MDA 8-OHdG Vitamin C TAS GSH GPx CAT SOD	↑ 8-OHdG in T2DM with micro- and macroalbuminuria compared to normoalbuminuric patients ↓ vitamin C in T2DM with micro- and macroalbuminuria compared to normoalbuminuric patients No differences in AGE, MDA, TAS, GSH, GPx, CAT, and SOD	Distributions were comparable in the three groups for aspirin and drugs for diabetes control (four kinds of medications) A lower percentage of T2DM without nephropathy used insulin and antihyperlipidemic drugs than T2DM with severe nephropathy A higher percentage of T2DM with mild nephropathy used metformin than the other two groups	[73]
T2DM with complications (*N* = 267) T2DM without complications (*N* = 366) Controls (*N* = 683)	Urine	8-OHdG 8-oxoGuo	↑ 8-OHdG and 8-oxoGuo in T2DM compared to controls ↑ 8-oxoGuo in T2DM with macrovascular complications compared to those without complications	Not provided	[91]
T2DM with microvascular complications (*N* = 30) T2DM without complications (*N* = 24) Controls (*N* = 22)	Urine	8-OHdG	↑ 8-OHdG in T2DM with microvascular complications than those without complications	Not provided	[93]
T2DM with microvascular complications (*N* = 25) T2DM with macrovascular complications (*N* = 25) T2DM without complications (*N* = 25)	Serum	AGEs Protein carbonyl MDA AOPP	↑ MDA, protein carbonyl, AOPP, and AGE in T2DM with micro- and macrovascular complications compared to T2DM without complications	Not provided	[64]
T2DM with micro-and macrovascular complications (*N* = 55) T2DM without complications (*N* = 26) Controls (*N* = 20)	Plasma	AGEs AOPPs	↑ AGE and AOPPs in T2DM compared to controls ↑ AGE in T2DM with complications compared to those without	No antioxidant supplements Lipid- or triglyceride-lowering drugs: 62% of T2DM without complications and 73% with complications	[76]
T2DM (*N* = 153) Controls (*N* = 65)	Plasma	AOPP	↑ AOPP in T2DM with albuminuria compared to those without AOPP was better than IMA in distinguishing patients with micro- and macroalbuminuria	Not provided	[85]
Newly diagnosed T2DM without albuminuria (*N* = 112) Newly diagnosed T2DM with albuminuria (*N* = 35) Controls (*N* = 49)	Plasma	AOPP	↑ AOPP in T2DM with albuminuria compared to those without and to controls	No hypoglycaemic or antihypertensive drugs, lipid-lowering agents, or antioxidants (vitamin C, vitamin E, or lipoic acid)	[86]
Poorly controlled T2DM with vascular complication (*N* = 44) Poorly controlled T2DM without vascular complication (*N* = 44) Controls (*N* = 31)	Plasma	AGE	↑ AGE in T2DM with complications compared to those without	Not provided	[77]
T2DM with nephropathy (*N* = 30) T2DM without nephropathy (*N* = 34) Controls (*N* = 20)	Serum and erythrocytes	MDA SOD	↑ MDA in T2DM compared to controls No changes in MDA and SOD in T2DM with and without nephropathy	Distributions were comparable in the groups with and without nephropathy for antihypertensives, statins, metformin, sulfonylureas, and insulin	[72]
T2DM with stable ischemic heart disease (*N* = 30) T2DM without stable ischemic heart disease (*N* = 30)	Plasma and serum	Protein carbonyl MDA	↑ MDA in T2DM with stable ischemic heart disease No differences in protein carbonyl	No antioxidants Distributions were comparable in the groups with and without ischemic heart disease for Ca channel blockers, beta blockers, ACE inhibitors/ARB, statins, and acetylsalicylic acid	[70]
T2DM with retinopathy (*N* = 45) T2DM without retinopathy (*N* = 19) Controls (*N* = 20)	Red blood cells	Protein carbonyl	↑ protein carbonyl in T2DM with retinopathy compared to those without and to controls	No vitamin E or C supplementation	[87]
T2DM with normoalbuminuria (*N* = 28) T2DM with microalbuminuria (*N* = 28) T2DM with macroalbuminuria (*N* = 28) Controls (*N* = 28)	Urine	HO1	↑ HO1/creatinine in T2DM with microalbuminuria and macroalbuminuria compared to those with normoalbuminuria and control ↑ HO1/creatinine in T2DM with normoalbuminuria than controls	Not provided	[111]
T2DM with retinopathy (*N* = 25) T2DM without retinopathy (*N* = 29) Healthy control (*N* = 24)	Serum	PON1	↑ PON1 in controls compared to T2DM ↑ PON1 in T2DM without retinopathy compared to T2DM with retinopathy	No patient under lipid-lowering therapy No patient was taking vitamin or mineral supplements or food fortified with vitamins	[105]
T2DM with macrovascular complications (*N* = 130) T2DM without complications (*N* = 135) Controls (*N* = 171) Ethnicity: Indians	Serum	PON1 AGEs	↑ PON1 in controls compared to T2DM ↑ PON1 in T2DM without complications compared to T2DM with macrovascular complications ↑ AGE in T2DM compared to controls ↑ AGE in T2DM with complications compared to those without	No antioxidants Distribution of antihypertensive and lipid-lowering drugs not provided	[106]

8-iso-PGF2*α*: 8-iso- prostaglandin F2*α*; 8-OHdG: 8-hydroxy-2′-deoxyguanosine; 8-oxoGuo: 8-oxo-7,8-dihydroguanosine; AGEs: advanced glycation end products; AOPP: advanced oxidation protein products;CAT: catalase; FRAP: ferric reducing ability of plasma; GSH: reduced glutathione; GSSG: oxidized glutathione; GPx: glutathione peroxidase; GR: glutathione reductase; HNE: 4-hydroxy-2-nonenal; HO1: heme oxygenase; MDA: malondialdehyde; PON1: paraoxonase 1; SOD: superoxide dismutase; TBARS: thiobarbituric acid reactive substances; TAS: total antioxidant status.

**Table 3:** Selected clinical prospective studies on circulating oxidative stress markers in T2DM. Categories are based on the following evidence levels: (A) large prospective studies and (B) prospective studies.

Disease and population	Sample	Markers	Observation	Class of evidence	Information on medication or supplements	Reference
T2DM (*N* = 1468)	Plasma	AOPP AGE Protein carbonyl Total reductive capacity of plasma	AOPP and total reductive capacity of plasma were not associated with the risk of major adverse cardiovascular events ↑ AGE and carbonyls were associated with the risk of major adverse cardiovascular events in univariate analysis but not in multivariate analysis	A	Use of statins included in the multivariate model	[80]
T2DM (*N* = 3766)	Urine	8-OHdG	↑ 8-OHdG is independently associated with all-cause mortality and cardiovascular mortality	A	Use of aspirin, statins, beta blockers, and ACE inhibitors/ARB included in statistical analysis	[95]
T2DM (*N* = 411)	Plasma	AGEs	Baseline AGEs associated with subclinical atherosclerosis parameters over 10 years of follow-up	B	No clear indication	[78]
T2DM (*N* = 1900)	Urine	8-oxoGuo 8-OHdG	8-oxoGuo was associated with all-cause mortality and cardiovascular death 8-OHdG was not associated with survival	A	No clear indication whether drugs were included in statistical analysis	[96]
T2DM (*N* = 1381)	Urine	8-oxoGuo	8-oxoGuo was associated with all-cause mortality and CVD at diagnosis and at 6-year follow-up	A	No clear indication	[97]
Newly diagnosed T2DM (*N* = 1381)	Urine	8-oxoGuo 8-OHdG	8-oxoGuo at diagnosis predicted all-cause and diabetes-related mortality over 6 years 8-OHdG was not associated with survival	A	No clear indication	[94]
T2DM (*N* = 716)	Plasma	AGEs	AGEs were associated with incident cardiovascular events over 3–7 years of follow-up	B	No clear indication	[79]
T2DM (*N* = 1412)	Plasma	AGEs Protein carbonyls	No independent associations between lower-extremity artery disease and AGE or protein carbonyls	A	Use of insulin therapy and antihypertensive,statin, fibrate, and antiplatelet drugs included in statistical analysis	[81]
T2DM (*N* = 108)	Serum	PON1	Lower PON1 activity and concentration were associated with an increased risk of developing cardiovascular disease	B	Not provided	[107]
(*N* = 234) with no preexisting diabetes	Serum	PON1	Lower PON1 activity and concentration were associated with an increased risk of developing T2DM	B	Not provided	[108]
(*N* = 5947) with no preexisting diabetes	Serum	PON1	Incident T2DM was not associated with PON1	A	Not provided	[109]

8-OHdG: 8-hydroxy-2′-deoxyguanosine; 8-oxoGuo: 8-oxo-7,8-dihydroguanosine; AGEs: advanced glycation end products; AOPP: advanced oxidation protein products; IMA: ischemia-modified albumin; PON1: paraoxonase 1.
